# A Coral Reef Algorithm Based on Learning Automata for the Coverage Control Problem of Heterogeneous Directional Sensor Networks

**DOI:** 10.3390/s151229820

**Published:** 2015-12-04

**Authors:** Ming Li, Chunyan Miao, Cyril Leung

**Affiliations:** 1 Detection and Control of Integrated Systems Engineering Laboratory in Chongqing Technology and Business University, Chongqing 400067, China; 2 School of Computer Science and Information Engineering, Chongqing Technology and Business University, Chongqing 400067, China; 3 School of Computer Engineering, Nanyang Technological University, Nanyang Avenue 639798, Singapore; ascymiao@ntu.edu.sg; 4 Department of Electrical and Computer Engineering, The University of British Columbia, Vancouver, BC V6T 1Z4, Canada; cleung@ntu.edu.sg

**Keywords:** directional sensor network, coverage control, coral reef algorithm, learning automata, multi-objective optimization

## Abstract

Coverage control is one of the most fundamental issues in directional sensor networks. In this paper, the coverage optimization problem in a directional sensor network is formulated as a multi-objective optimization problem. It takes into account the coverage rate of the network, the number of working sensor nodes and the connectivity of the network. The coverage problem considered in this paper is characterized by the geographical irregularity of the sensed events and heterogeneity of the sensor nodes in terms of sensing radius, field of angle and communication radius. To solve this multi-objective problem, we introduce a learning automata-based coral reef algorithm for adaptive parameter selection and use a novel Tchebycheff decomposition method to decompose the multi-objective problem into a single-objective problem. Simulation results show the consistent superiority of the proposed algorithm over alternative approaches.

## 1. Introduction

In recent years, with the development of micro-electromechanical systems (MEMS) directional sensor networks (DSNs) have received much attention due to their wide and significant applications [1], which offer important economic benefits. A DSN is a wireless network which is equipped with directional sensors such as video sensors, ultrasound and infrared sensors. Differing from the sensor nodes of the traditional wireless sensor networks (WSN) which have omni-directional sensing ranges, the sensing range of DSN sensor nodes are restricted by their directions and specific angular dimensions.

Since directional sensing and directional communication have great impact on the performance of a DSN, several difficulties have emerged in DSN protocol design. One of the most fundamental problems is the coverage issue. Although there are extensive amounts of research about the coverage problems in WSNs, however, these WSN research results cannot be directly applied to DSNs. 

In this paper, a coverage control algorithm based on a multi-objective optimization method is proposed to reduce the power consumption of networks and prolong the lifetime of the network. It should be noted that two assumptions which are generally considered in the published papers about the coverage problems of DSNs are relaxed in order to formulate a more realistic DSN deployment problem. The first assumption concerns the types of sensor nodes in the DSN. Indeed, in most research works, sensor nodes are identical in terms of energy and hardware complexity. We consider in the paper a heterogeneous directional sensor network (HDSN), in which each node has different sensing radius, communication radius and field of angle. To the best of our knowledge, research on the network coverage in HDSNs is less evident. The second assumption is the sensing requirement within the monitored area. In most of the published papers, the whole supervised region is considered to be of the same importance. In other words, the sensing requirement is uniformly distributed within the area [2]. However, this assumption is not always valid for some applications. For example, in water quality monitoring, the risky area near the chemical deposits or animal/human habitats needs high detection levels, while for other areas a low detection level is sufficient. To sum up, the key contributions of this paper are as follows:

- Firstly, we propose a realistic case study of a coverage algorithm for the placement field characterized by a geographical irregularity of the sensed events in a HDSN by ensuring the connectivity of the network, reducing the cost of deployment.
- Secondly, this paper proposes a learning automata-based coral reef algorithm for adaptive parameter selection. Learning capabilities are used in the coral reef algorithm to select its parameters. As a result, the convergence rate and the search abilities of the algorithm are enhanced. Experimental results show the superiority of the proposed algorithm.
- Thirdly, a novel Tchebycheff decomposition approach is introduced to decompose the multi-objective problem into a single-objective problem. Theoretical proofs and numerical results indicate the efficiency of the proposed method.

The rest of the paper is organized as follows: Section 2 reviews some related works on DSN deployment issues. Mathematical formulation of the coverage model in HDSN is formalized in Section 3. In Section 4 and Section 5, fundamentals of the coral reef algorithm and learning automata are introduced, respectively. An improved Tchebycheff decomposition method and the proposed learning automata- based coral reef algorithm are detailed in Section 6. The experimental results of the proposed approaches are given in Section 7. Finally, Section 8 ends the paper with some concluding remarks and future research directions. 

## 2. Previous Works

As some of the most promising tools, multi-objective optimization algorithms have been applied to wireless sensor networks to balance various trade-offs among different conflicting objectives. A detailed survey can be found in [3]. By decomposing the multi-objective optimization problem into a set of single objective subproblems which are tackled in parallel, the Multi-objective Evolutionary Algorithm based on Decomposition (MOEA/D) [4] has been used in coverage optimization problems relating to WSNs. In [5], the authors proposed a generalized subproblem-dependent heuristic (GSH) and successfully hybridized it with MOEA/D for tackling the multi-objective dense deployment and power assignment problem in WSNs. In [6], the authors deal with how to efficiently deploy energy-harvesting relay nodes in a WSN. However, it should be noted that the weighted Tchebycheff Approach used in the above two papers is very sensitive to the scale of the objectives. To correct this situation an enhanced version of the Tchebycheff Approach is proposed in this paper.

The parameter settings of the evolutionary algorithm have a great impact on the performance of the algorithm. The self-adaptive parameters adjustment method is an effective way to improve the diversity of the individuals and avoid premature convergence. Since learning automata are adaptive decision making devices that run in an unknown environment and progressively enhance their performance via a learning process, it has been proved that the method that combines the learning ability of the learning automata with the parameter adjustment of the evolutionary algorithm will enhance the efficiency of the evolutionary algorithm [7,8]. Inspired by this idea, a learning automata-based evolutionary algorithm is proposed to solve our multi-objective problem.

In recent years, a considerable amount of work on coverage in directional sensor networks (DSNs) has been reported in the literature [9]. In [10], the authors designed two greedy-based scheduling algorithms that aim to select the appropriated sensor direction and sensing range in a way to meet the requirements of the target coverage problem and at the same time maximize the network lifetime. However, the performance of the algorithms is extremely dependent on the closeness of the initial candidates to the optimal solution. In [11,12,13,14], they all aimed to maximize the network lifetime by finding cover sets in each of which the directions cover all the targets. In [15] and [16], the authors utilized the geometrical features of a Voronoi diagram to propose a distributed Voronoi-based self-redeployment algorithm aiming to improve the overall field coverage of directional sensor networks. In [17], the authors proposed heuristic algorithms to solve the multiple directional cover set problem. To model an application scene more accurately, Ma et al. have proposed a 3D sensor coverage-control model with tunable orientations [18]. In [19], learning automata algorithms are employed to find a near-optimal solution for solving the target coverage problem in DSNs. However, it should be noted that all the abovementioned algorithms assume that the coverage requirement is uniformly distributed within the area, therefore these algorithms cannot perform well in real scenarios characterized by the geographical irregularity of the sensed events. To solve such a problem, several algorithms were proposed in [20,21,22]. In [20], Wang *et al.* proposed to choose a minimum subset of directional sensors that is able to satisfy the prescribed priorities of all the targets. In [21], the authors proposed a greedy-based scheduling algorithm to find a sequence of feasible cover sets in order to prolong the network lifetime. In [22], the authors propose a learning automata-based algorithm to organize the directional sensors into several cover sets in such a way that each cover set can satisfy coverage quality requirement of all the targets.

While coverage maintenance protocols for DSNs have been extensively studied in the literature, no attempt, however, has been made on tackling coverage problems, energy consumption and connectivity problems for DSNs, especially for DSNs with heterogeneous nodes in terms of field of angle view, sensing and communication radius. To this end, a multi-objective optimization-based coverage control algorithm for HDSNs is proposed to deal with such problems in HDSNs. As the performance of this algorithm is sensitive to the proper setting of the parameters, the learning capabilities of the learning automata are employed for adaptive parameter selection. Moreover, an improved Tchebycheff decomposition method is proposed to assess the solutions obtained from the evolutionary algorithm, which can provide a collection of non-dominant solutions in a single run. Besides, to our knowledge, no attempt has adopted multi-objective evolutionary algorithms for coverage and efficient design issues in HDSNs on the condition of the geographical irregularity of the sensed events. Thus, in this paper we deal with the development of an evolutionary algorithm-based coverage control protocol that efficiently sets up the minimum number of active sensors, while satisfying the coverage and connectivity requirements. Consequently, unwanted interference at the MAC layer is avoided and energy consumption is reduced. The characteristics optimized by the evolutionary algorithm in this paper include network coverage, network connectivity and the status of the sensor nodes (whether they are active or inactive). 

## 3. Problem Statements

This section first demonstrates the system models and assumptions based upon which the problem is formulated, and then presents the problem formulation. 

### 3.1. System Model and Assumptions

The system model and general assumptions are as follows:

(1) There is a rectangular monitoring area *A* in which the sensors are placed. In order to reduce computational complexity of the problem, *A* is divided into *m* × *n* uniform consecutive small grids and each size of grid is 1.
(2) A DSN is formed by *N* heterogeneous directional sensors *s_i_*(*i* = 1, 2, …, *N*) in terms of the sensing radius, communication radius and field angles of view.
(3) The directional sensor nodes are scattered randomly and uniformly within the field. Each sensor has only one sensing radius and sensing direction. All the sensors are static after deployment and their locations are known.
(4) If a sensor *S_a_* is within the communication radius of a sensor *S_b_*, then *S_b_* can communicate with sensor *S_a_*.
(5) A target can be sensed by a sensor node if the target is located within the sensing radius and field of view of the node.
(6) The geographical distribution of the sensing events or targets is supposed to be non-uniformly distributed within the zone. That is to say, the subareas of the monitoring area are not of the same importance. The area where targets or monitoring events appear frequently is called a hot spot area.

### 3.2. Problem Formulation

The objective of the coverage control algorithm is to obtain a set of sensor nodes that fulfill the following optimization criteria:

(1) Maximize the coverage rate of area. Since the targets are usually non-uniformly distributed in the area, more sensors are needed in some subareas where the targets tend to be clustered around to enhance the quality of service for the WSN and fault tolerance of the network. Based upon this, we need more sensors to cover any hot spots of the supervised area (often called *k*-coverage), and in the remaining area, we only need to guarantee that there are some sensors which can monitor it. In the mathematical description, the coverage rate of the area is defined as the percentage of the covered grids over the total grids of *A*. It is evaluated as follows: 

Max:

$$f_{1}=\frac{\sum_{(x,y)\in A}c(x,y)}{W}$$

where *W* is the number of grids in surveillance zone *A* and *c*(*x*, *y*) is calculated using following equation:

$$c(x,y)=\left\{ \begin{array}{c} 1\;\mathrm{grid}(x,y)\;\mathrm{is}\;\mathrm{achieved}\;\mathrm{coverage}\;\mathrm{requirement}\\ 0 \end{array}\right.$$

where grid (*x*, *y*) is achieved coverage requirement means if the grid (*x*, *y*) is located within a hot spot subarea, the coverage requirement is *K*-coverage (*K* > 1); otherwise, the coverage requirement is covered by one sensor node.

(2) Minimize the number of working sensors nodes, or equivalently, minimize the financial cost of working nodes, which is defined as:

Min:

$$f_{2}=\frac{\left|\sum_{s_{i}\in S^{\prime }}s_{i}\right|}{N}$$

where *S*’ is set of working sensors, and |*s_i_*| is evaluated in the following way:

$$\left|s_{i}\right|=\left\{ \begin{array}{l} 1\;s_{i}\in S^{\prime }\\ 0\;\mathrm{otherwise} \end{array}\right.$$

(3) Maximize the connection status of working nodes, *i.e.* for each node *s_i_* (*i* = 1, 2,…, *N*) belonging to a set of *S*’, if *s_i_* is located in the hot area, *K*-connect (*K* > 1) is necessary for network reliability and fault tolerant; otherwise, in the rest of the monitor area, that is non-hot area*, s_i_* can connect another sensor in the set of *S*’, that is OK. Connect status of working nodes is denoted as:

Max:

$$f_{3}=\frac{\left|\sum_{s_{i}\in S^{\prime }}Cn(s_{i})\right|}{\left|S^{\prime }\right|}$$

where *Cn*(*s_i_*) is the connect status of sensor *s_i_* and is defined as:

$$Cn(s_{i})=\left\{ \begin{array}{l} 1\;s_{i}\;\mathrm{located}\;\mathrm{within}\;\mathrm{hotspot}\;\mathrm{region}\;\mathrm{and}\;{\text{s}}_{\text{i}}\;\mathrm{is}\;k-\mathrm{connected}\;(k>1)\\ 1\;s_{i}\;\mathrm{located}\;\mathrm{at}\;\mathrm{non}-\mathrm{hotspot}\;\mathrm{region}\;\mathrm{and}\;{\text{s}}_{\text{i}}\;\mathrm{is}\;1-\mathrm{connected}\\ 0\;\mathrm{otherwise} \end{array}\right.$$

In the above formula, if *s_i_* is located within the hotspot region and it is *k*-connected (that means it has more than one sensor within its communication radius), then its value of *C_n_*(*s_i_*) is 1. If *s_i_* is placed within the non-hotspot region and it has only one sensor to directly communicate with, then its value of *C_n_*(*s_i_*) is 1. Except for the cases presented, the value of *Cn*(*s_i_*) is 0.

The problem we aim to solve is multi-objective optimization, which can be described as follows:

$$F=(\max(f_{1}),\;\min(f_{2}),\;\max(f_{3}))$$

The problem presented above is a *NP*-hard problem [23]. Its solution space is as large as 2*^N^*. To resolve such a combinatorial optimization problem, it is difficult to use an exact method as Branch & Bound due to its exponential complexity. In this paper we use a bio-inspired algorithm to solve this multi-objective optimization problem. 

## 4. Original Coral Reef Optimization Algorithm

The coral reef optimization (CRO) algorithm is a novel bio-inspired algorithm for solving optimization problems, which mimics the behaviors of corals’ reproduction and coral reef formation. It was originally introduced by Salcedo and his colleagues [24] and thereafter has found a lot of applications in the optimization of mobile network deployment problems [25], global solar radiation prediction [26], wind speed prediction [27,28], *etc*. Algorithm 1 summarizes the skeleton of the CRO algorithm applied to a combinatorial optimization problem for minimizing one objective function.

**Algorithm 1** The CRO algorithm1. Initialize the reef2.While not stopCriteria () do {3. A fraction of *p_k_* coral larvae formed by external reproduction(also called broadcast spawning)4. The rest of (1-*p_k_*) coral larvae formed by internal sexual reproduction( brooding )5. Larvae setting procedure6. Asexual reproduction7. Depredation in polyp phase8. End while }9.Return solution to the problem

In the CRO implementation there are two main phases [28]: 

Phase 1: Initialization of CRO parameters. 

The purpose of CRO initialization is to set parameters to fill in the algorithm. The main control parameters of CRO are presented as follows: coral reef, Λ, consisting of a *T* × *M* grid similar to the population size in the evolutionary algorithm (EA), the girds are selected randomly and can assign a coral or colony of coral, representing a solution to the given problem, the rate *ρ_0_* between the selected grids and not selected ones which is an important factor to control the exploration ability of the algorithm. The health function *f* is similar to the fitness of EA. The underlying idea behind CRO is that as the reef progresses, the more healthy the corals are (which represents a better solution to the mentioned problem), the better the chance they can survive.

Phase 2: reef formation.

The reef formation consists of the following steps:

(1) Broadcast Spawning (external sexual reproduction):

- Choose a fraction of the existing corals *p_k_* to become broadcast spawners. The remaining ones will be formed by internal sexual reproduction.
- Broadcast spawner couples will produce a coral larva by sexual crossover. It should be noted that once two corals have been selected to be the parents of a larvae, they are not selected any more in Broadcast Spawning phase.

(2) Brooding (internal sexual reproduction) 

As Step (1) describes, the fraction 1- *p_k_* of corals will form in this stage by means of a random mutation. The newly produced larvae together with that of larvae formed in Step (1) are released to the water.

(3) Larvae setting 

When all the larvae are formed by broadcast spawning or by brooding, the process of setting and growing in the reef will begin. The health function of each larva is evaluated firstly. Then, each larvae will settle down in the grid (*i, j*) of the reef randomly. If the grid is free space in the reef, the coral can grow whatever its value of health function. However, if the grid is already occupied by a coral, the new larvae will set in the occupied grid only when its health function is better than the existing one. A variable *κ* is defined to represent the maximum number of attempts for the larvae to set in the reef before it is preyed on by other animals.

(4) Asexual reproduction 

According to the values of health function, the existing corals in the reef are rearranged. A proportion *F_a_* of the existing corals will copy itself and attempt to settle in a different part of reef by Step (3) introduced above.

(5) Depredation in polyp phase 

In the process of reef formulation, some corals may die. As a result, space is freed up for the newly generated corals. The probability of the depredation process is *F_d_*, and only be applicable for a proportion *F_d_* of coals with the worst health function values. It should be noted that, *F_a+_* × *F_d_* ≤ 1, that is to say, no overlap between the coral set formed by Step (4) and depredated by Step (5).

## 5. Learning Automata

A learning automaton [29] is considered as an adaptive decision making unit that is continually interacting with the environment to learn how to choose an optimal action from its action set. The action selected by the learning automaton is evaluated by the environment and a reinforcement signal will return as a feedback for the selected action. The automaton uses the feedback to update its action probability vector set. By continuing the above process, the optimal action from the action-set will be found and the average reward feedback signals received from the environment could be maximized [30,31]. The four-tuple {α, β, *P*, *T*} can be used to represent a learning automaton, where $\alpha =\{\alpha_{1},\alpha_{2},\cdots ,\alpha_{r}\}$ is a set of available actions which can be selected by the learning automaton. $\beta =\{\beta_{1},\beta_{2},\cdots ,\beta_{r}\}$ signifies the set of the potential values of the reinforcement signal, $p=\{p_{1},p_{2},\cdots ,p_{r}\}$ is a state probability vector and each *p_r_* associated with the selected action *α_r_*, *T* is a learning algorithm used to update the state probability vector.

In the basic learning algorithm, named Linear Reward Penalty Algorithm, the action probability vector is updated applying to Equation (1) for favorable responses and Equation (2) otherwise:

(1) $$p_{i}(n+1)=p_{i}(n)+\alpha [1-p_{i}(n)]\;p_{j}(n+1)=(1-\alpha )p_{j}(n)\;\forall j,j\neq i$$

(2) $$p_{i}(n+1)=(1-b)p_{i}(n)\;p_{j}(n+1)=\frac{b}{r-1}+(1-b)p_{j}(n)\;\forall j,j\neq i$$

where *r* is the cardinality of the action set, *a* and *b* are the reward and penalty parameters, respectively.

## 6. The Proposed Algorithm

This section details a novel fitness evaluation method for the multi-objective optimization problem and the proposed learning automata-based coral reefs algorithm, respectively. It should be noted that the underlying idea behind the proposed method might shed light on the design of another heuristic method for multi-objective optimization problems.

### 6.1. Proposed Tchebycheff Decomposition Approach 

In this subsection, a newly improved multi-objective optimization fitness evaluation method based on the Tchebycheff decomposition approach is proposed. Firstly, a few concepts for the multi-objective optimization are defined to facilitate our presentation. A general multi-objective optimization problem is described as [32]:

Min:

(3) $$\begin{array}{l} F(x)=((f_{1}(x),f_{2}(x),\ldots ,f_{t}(x)){)}^{T}\\ s.t.\;x\in S,\;x=((x_{1},x_{2},\ldots ,x_{n}){)}^{n} \end{array}$$

where *f*_1_(*x*), *f*_2_(*x*),…,*ft*(*x*) are the *t* objective functions, (*x*_1_,*x*_2_,…*x_n_*) are the *n* optimization parameters and j∈{1,2,…,t} *S* ∈ *R^n^* is the solution space. The definition of Pareto dominance and Pareto optimality are presented as follows:

**Definition 1.**  ***(Pareto dominance)** [33]: Suppose x and y are two decision variables, x is said to dominate y, denoted by x≻y, if and only if $f_{i}(x)\geq f_{i}(y)$ for every i∈{1,2,…,t} and $f_{j}(x)>f_{j}(y)$ for at least one index j∈{1,2,…,t}*.

**Definition 2.**  **(Pareto optimality)** [33]: $x^{*}\in S$ is said to be Pareto optimal (or non-dominated) if there is no another x∈S so that x dominates x*. The set of all Pareto optimal solutions in the decision space is called the Pareto optimal Set (PS).

An improved fitness evaluation based on Tchebycheff decomposition approach is:

$$\min\;T_{s}(f(x)|(\lambda^{i},y^{*},y^{\#}))=\underset{1\leq j\leq t}{\max}\;\{\lambda_{j}^{i}(\frac{f_{j}(x)-y^{*}}{y^{\#}-y^{*}})\}\;(\text{A})$$

where $\lambda^{i}$, $y^{*}$, $y^{\#}$ are constant values; $\lambda^{i}=(\lambda_{1}^{i},\lambda_{2}^{i},\ldots ,\lambda_{t}^{i})$ is the aggregation coefficient vector, $\sum_{j=1}^{t}\lambda_{j}^{i}=1;\;$$y^{*}=(y_{1}^{*},y_{2}^{*},\ldots ,y_{t}^{*})$ and $\;y^{\#}=(y_{1}^{\#},y_{2}^{\#},\ldots ,y_{t}^{\#})$ are the lower bounds and upper bounds of the objective functions, $\forall j,\;j\in \{1,2,\ldots ,t\},y_{j}^{*}=\min\{f_{j}(x)\},\;y_{j}^{\#}=\max\{f_{j}(x)\}$. $y^{*}$ and $y^{\#}$ are introduced to compare the values of the different objective functions. 

In the improved Tchebycheff approach, let $\lambda^{1},\ldots ,\lambda^{W}$ be the aggregation coefficient vectors, subproblem *i* corresponds to the coefficient vector, where $\lambda_{j}^{i}\geq 0,j=1,\ldots ,t,\;\sum_{j=1}^{t}\lambda_{j}^{i}=1,i=1,\ldots ,W$. *t* is the number of objectives, and *W* is the number of subproblems. Population size *W* (*i.e*., the number of subproblems) and aggregation coefficient vectors $\lambda^{1},\ldots ,\lambda^{W}$ are controlled by *t* and an integer *H*. More precisely, $\lambda^{1},\ldots ,\lambda^{W}$ are all the weight vectors in which each individual weight takes a value from {0/*H*, 1/*H*,…,*H*/*H*}. Therefore, the number of such vectors is $W=C_{H+t-1}^{t-1}$.

We use Formula (A) to change them into the same range of value in order to make comparisons. The proof of Proposition (A) will be given in Theorem 2.

**Lemma:**  *for* $\forall y^{1},y^{2}\in R^{n},\;\mathrm{if}\;y_{j}^{1}\leq y_{j}^{2}\;\mathrm{and}\;y^{1}\neq y^{2}\;\mathrm{for}\;\mathrm{all}\;j(\;j=1,2,\ldots ,t)$ *, then* $T_{s}(y^{1})<T_{s}(y^{2})$*.*

**Proof:**  Let us take *y*^1^, *y*^2^ into the Formula (A), note that the two parts in the right side of the equation for the Formula (A) are positive. Therefore, it is easy to get the conclusion that $T_{s}(y^{1})<T_{s}(y^{2})$.☐

**Theorem 1.**  If x* is an optimal solution for the single objective Problem (A), then x* is the Pareto optimal solution for the Problem (1).

**Proof:**  We will prove it by the contraction method. Suppose x* is not Pareto optimal solution to the Problem (1), according to Definition 2, $\exists x^{\prime }\;f(x^{\prime })≺f(x^{*})$. From Definition 1, we know, $\forall j\in \{1,2,\ldots ,n\},\;f_{j}(x^{\prime })\leq f_{j}(x^{*})$ and $f_{j}(x^{\prime })\neq f_{j}(x^{*})$. From the Lemma, we can get $T_{s}(f_{j}(x^{\prime }))<T_{s}(f_{j}(x^{*}))$, that is to say, x* is not an optimal solution to the Problem (A). Therefore, it apparently contradicts the hypothesis that x* is the single objective Problem (A). ☐

**Theorem 2.**  If x* is a Pareto optimal solution to Problem (1), then $\exists \lambda^{*}=(\lambda_{1}^{*},\lambda_{2}^{*},\ldots ,\lambda_{t}^{*})$ makes $T_{s}(f(x^{*}))\leq T_{s}(f(x))$, wherein $\forall \lambda_{j}^{*}\geq 0,\;\sum_{j=1}^{t}\lambda_{j}^{*}=1$.

**Proof:**  We will prove it by the contraction method. Suppose that $\exists \lambda^{*}\;T_{s}(f(x^{*}))\leq T_{s}(f(x))$. In other words, for ∀λ $\lambda =(\lambda_{1},\lambda_{2},\ldots ,\lambda_{n}),\;\exists x^{\prime },\;T_{s}(f(x^{\prime }))<T_{s}(f(x^{*}))$, where $\forall \lambda_{i}\geq 0,\;\sum_{i=1}^{t}\lambda_{i}=1$. By Formula (A), and thus $\forall j,\;f_{j}(x^{\prime })<f_{j}(x^{*})$, that is to say $f(x^{\prime })≺f(x^{*})$. Therefore, it apparently contradicts the hypothesis. ☐

From the theorems presented above, we can see that the optimal solutions to the single objective function optimization Problem (A) are the Pareto optimal solutions to the multi-objective function. By using Theorem 2, a multi-objective function optimization problem can be changed into a single-objective function optimization problem. 

### 6.2. Proposed Hybrid Learning Automata—CRO Algorithm

The coral reef algorithm described in Section 4 shows that the parameters of CRO, that is, Broadcast Spawning radio *F_b_* and brooding radio 1-*F_b_* play important roles in the exploration/exploitation capabilities of the algorithm. In this subsection a new learning automata mechanism for adaptive parameter selection for the CRO is given.

**Figure 1:**
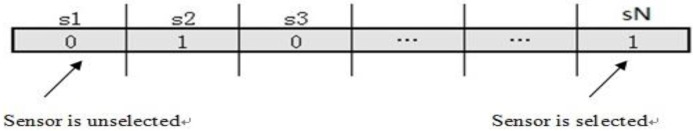
Solution representation in LACRO.

The proposed algorithm, that is the learning automata based coral reefs optimization algorithm (LACRO for short), performs like the original CRO algorithm with the auxiliary section at the end of the each iteration to select CRO parameters value. In order to enhance the ability of the original CRO algorithm, we borrow the idea from the differential evolution [34] and the genetic algorithm to make some modifications to the basic process of the CRO algorithm. The great difference between the original CRO algorithm and the proposed method lies in that the mutation of the selected brooding coral is controlled by the parameter *B_d_*, which is adaptive and adjusted by learning automata according by the diversity and evolutionary status of the population. In contrast to the proposed algorithm, the fraction of corals that reproduce by brooding is 1-*F_b_* in the original CRO. In addition to the parameter of the brooding radio controlled by the learning automata, the parameter of Broadcast Spawning radio *F_b_* is also adaptive and adjusted by the learning automata. The details of the proposed algorithm are presented as follows:

Step 1: Coral reefs initialization. Set the initial parameter values for the coral reef population, the learning automata and determine the way of encoding the solution. Considering the characteristics of the solved problem, we use the binary code method. A solution to the problem is demonstrated in Figure 1, where “1” stands for the sensor which is selected to be in the working status, otherwise, the bit is “0”.

Step 2: Equally discretize two parameters, namely, the value of *F_b_* and *B_d_*, into the *m*_1_ distant value and *m*_2_ distant value, respectively. This method is called the adventurous method [7] which allows a parameter to change radically from one end of its range to the other in the consecutive iterations and not to be restricted by its previous value. 

Step 3: To equip each parameter with one learning automaton *LA_i_* (*i* = *F_b_*, *B_d_*)_,_ in which the corresponding actions number is *m*_1_ and *m*_2_, respectively. During every iteration, *LA_i_* (*i* = *F_b_*, *B_d_*) chooses one from its action set, then the corresponding value of the selected action will be set as the new value for the parameter. In our proposed method, all the coral reefs have the same values for the parameter *F_b_* and *B_d_*. In each iteration the roulette-wheel selection method is used to select the corresponding action of each learning automaton. 

The pseudo codes for LACRO are listed in Algorithm 2:
**Algorithm 2**. Pseudocode for the proposed LACRO algorithm Input: parameters *F_b_* and *B_d_* , success threshold θ, the parameters of learning automata and CROOutput: The solutions to the problem1. Coral reefs initialization 2. Equally divide parameter *F_b_* into *m*_1_ parts and parameter *B_d_* into *m*_2_ parts3. Assign $LA_{F_{b}}$ with *m*_1_ actions to *F_b_* and the probability of each action is 1/ *m*_1_4. Assign $LA_{B_{d}}$ with *m*_2_ actions to *B_d_* and the probability of each action is 1/ *m*_2_5*. N* is the number of coral reefs and *N*’(*t*) is the number of coral reefs which their fitness has been improved since iteration *k*−16. While not stopCriteria () do {7. Using the roulette-wheel method to select Active Action $P_{F_{b}}$ for $LA_{F_{b}}$ and $p_{B_{d}}$ for $LA_{B_{d}}$8. Select the value of parameter *F_b_* and *B_d_* according to $p_{F_{b}}$ and $p_{B_{d}}$, assuming the selected value is *f_b_* and *b_d_* respectively9. A fraction of *f_b_* coral larvae formed by external reproduction(also called broadcast spawning)10. The rest of *b_d_* coral larvae formed by internal sexual reproduction( brooding )11. Fitness evaluation of newly produced coral larvae by broadcast spawning and brooding12. If $\frac{N\prime (t)}{N}\geq \theta$ {13. Reward the selected action for $LA_{F_{b}}$ and $LA_{B_{d}}$ and according to Equation (1)13. Reward the selected action for $LA_{F_{b}}$ and $LA_{B_{d}}$ according to Equation (1)14. Else15. Punish the selected action for $LA_{F_{b}}$ and $LA_{B_{d}}$ according to Equation (2) }16. Larvae setting procedure17. Asexual reproduction18. Depredation in polyp phase19. End while }

Step 4: Fitness evaluation and learning automata updating. Please see Subsection 6.1 for the detailed fitness evaluation method. The learning automata are updated according to the evaluation result of parameter selection. It is considered to be a “successful” selection on the condition that the fraction of improved coral reef in the previous iteration is greater than the specific threshold. In this case, the two learning automata are rewarded; otherwise they are penalized. As a result, the actions with greater probability stand a good chance of being selected. 

Step 5: Larvae setting process.

Step 6: Asexual reproduction process.

Step 7: Depredation in polyp phase.

Steps 5–7 are the same as described in Section 4. Please see the relevant parts for detailed information.

Step 8: To determine whether the goal of stopping criteria is achieved. If it is true, return the best solution, otherwise, return to Step 4.

## 7. Performance Evaluation

In this section, the performance of the proposed algorithm is investigated. Firstly, performance comparison in test functions including single-objective functions and multi-objective functions were made. Subsequently, the proposed algorithms were applied to solve the coverage problem of directional heterogeneous sensor network. All the algorithms were implemented using MATLAB 2013b and executed on a computer with a Core i5 2.3 GHz Quad CPU, 4 GB RAM and Windows 7 64 bit operating system.

### 7.1. Performance Evaluation in Test Functions 

#### 7.1.1. Single-Objective Function

Three well-studied test functions in benchmarking optimization algorithms, namely Rastrigrin, Rosenbrock, and Griewank are used to compare the performances of the LACRO and the original CRO algorithm. In all the simulation, the termination criterion is set to 800 iterations. The range of values for *F_b_* and *b_d_* are [0.75, 0.95] [35] and [0.5, 0.8] [28], respectively. The value of *m*_1_ and *m*_2_ are 5 and 6. That is to say, the values of *F_b_* and *b_d_* are selected by the learning automata from the set {0.75 0.8 0.85 0.9 0.95} and set {0.5 0.55 0.60 0.65 0.7 0.75 0.8}, respectively. The success threshold parameter *θ* is 0.8, and the population size is 300 (*H* = 23, *t* = 3, $W=C_{H+t-1}^{t-1}$ = 300). The parameters in the learning automata are *a* = *b*= 0.01 [8]. The parameter of the function dimension *D* is 30 and the details of the test functions are as follows:

(1) Rastrigrin $\min\;f_{1}(x)=\sum_{i=1}^{n}\left(x_{i}^{2}-10\cos(2\pi x_{i}))+10\;x_{i}\in [-5,5\right]$
(2) Rosenbrock $\min\;f_{2}(x)=\sum_{i=1}^{n}100{\left(x_{i+1}-x_{i}\right)}^{2}+{\left(1-x_{i}\right)}^{2}\;x_{i}\in [-100,100]$
(3) Griewank $\min\;f_{3}(x)=\frac{1}{4000}\sum_{i=1}^{n}x_{i}^{2}-\overset{n}{\underset{i=1}{\Pi }}\cos(\frac{x_{i}}{\sqrt{i}})+1\;x_{i}\in [-600,600]$

Every test function has its own characteristics that make it suitable for evaluating the efficiency of the proposed algorithm. Rastrigin and Griewank are multimodal functions with many local optima that increase in the exponential way with problem dimensions, while Rosenbrock is a unimodal function.

**Table 1:** Results on three functions based on 10 independent runs.

Function	LACRO	CRO
$f_{1}$	Mean 0.0531 SD 0.02257	Mean 0.0733 SD 0.0247
$f_{2}$	Mean 3.3884 SD 91.8525	Mean 28.4710 SD 0.7326
$f_{3}$	Mean 0.1344 SD 0.1446	Mean 0.2578 SD 0.15297

**Figure 2:**
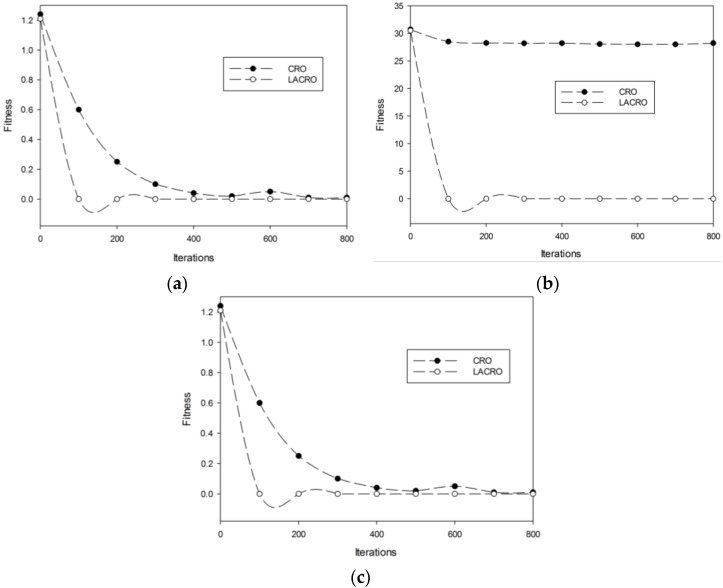
Comparison of LACRO and CRO. (**a**) *f*_1_ Rastrigrin; (**b**) *f_2_* Rosenbrock; (**c**) *f*_3_ Griewank.

Table 1 demonstrates the global mean values and the standard deviation (SD) of the final solutions during 10 rounds of simulation. The results in Table 1 show that the proposed LACRO algorithm obtains the goal of the best solutions for the testing functions. The comparisons prove that the mechanism of the learning automata-based parameters self–adaptive indeed makes the LACRO achieve better performance than the original CRO algorithm. It can effectively avoid local optima and premature convergence, finding optimal solution accuracy in test multimodal functions. Figure 2a–c illustrate the evolution of optimal fitness for two algorithms. As can be seen from Figure 2a–c, the proposed algorithm performs better and converses more rapidly than the original CRO algorithm. The proposed algorithm LACRO successfully gets the local optimal solution (0, 0… 0) in the end. Therefore, the proposed LACRO performs better than CRO in terms of convergence speed and final solution.

#### 7.1.2. Multi-Objective Function

To better verify the validity of the proposed fitness evaluation method and the performance of LACRO algorithm, we carried out a number of experiments on multi-objective function optimization. In this subsection, performance metrics for multi-objective optimization are firstly introduced. Then three test functions are presented. Finally, the results will be given at the end of this subsection.

(1) Performance metrics

In our experiments, the following performance index is used:

Set coverage(C-metric): The C-metric [36] evaluates the rate between dominance of a Pareto front over another Pareto front. Assuming that *A* and *B* are two approximated Pareto-optimal sets, the coverage of two sets *C*(*A*, *B*) is defined as the percentage of the solutions in *B* that are dominated by at least one solution in *A*, *i.e.*:

$$C(A,B)=\frac{\left|A-\{x\in A|\exists y\in B:y≺x\}\right|}{\left|A\right|}$$

The smallest *C*(*A*, *B*) is, the better the *A*. It should be noted that *C*(*A*, *B*) is not necessarily equal to 1 − *C*(*B*, *A*). *C*(*A*, *B*) = 1 means that all solutions in *B* are dominated by some solutions in *A*, while *C*(*A*, *B*) = 0 implies that no solution in *B* is dominated by a solution in *A*.

(2) Test functions

We use three widely used bio-objective test instances [37,38,39] to compare LACRO with the original CRO. All these test instances are minimization of the objectives. They are selected based on following properties: (i) the maximum value and minimum value of each sub problem in the test functions is easy to obtain; (ii) Exact shape and location of resulting PF for these problems are known. Table 2 presents the details of the test problems used in the paper. The experiment environment is the same as the performance simulation in single objective function section.

**Table 2:** Test problems used in this study.

Problem	Objective Functions	Domain
F1	$\begin{array}{c} f_{1}=x^{2}\\ f_{2}={\left(x-2\right)}^{2} \end{array}$	[−5, 7]
F2	$\begin{array}{c} f_{1}(x,y)={\left(x^{2}+y^{2}\right)}^{\frac{1}{8}}\\ f_{2}(x,y)={\left[{\left(x-0.5\right)}^{2}+{\left(y-0.5\right)}^{2}\right]}^{\frac{1}{4}} \end{array}$	[−5, 10]
F3	$\begin{array}{c} f_{1}(x)=\left\{ \begin{array}{l} -x\;x\leq 1\\ -2+x\;1<x\leq 3\\ 4-x\;3<x\leq 4\\ -4+x\;x>4 \end{array}\right.\\ f_{2}(x)={\left(x-5\right)}^{2} \end{array}$	[−5, 10]

(3) Results

The parameter setting of the algorithm is the same as the single-objective test section. In formula (A), each individual weight $\lambda_{1},\lambda_{2},\ldots ,\lambda_{W}$ (*W* is the population size) takes a value from the set of {0/300, 1/300, … , 300/300}. Table 3 describes of the C-metric values of the final approximations obtained by LACRO and original CRO. The values in the brackets are the standard deviation of the solutions. As can be seen from Table 3, LACRO performs better than CRO in terms of C-metric. Taking Function F1 as an example, on average, 97% of the final solutions generated by CRO are dominated by LACRO and 89% vice versa.

**Table 3:** Average value of C-metric based on 10 independent runs.

Function	C (LACRO, CRO)	C (CRO, LACRO)
F1	0.97 (0, 00005)	0.89 (0, 00042)
F2	0.59 (0, 00008)	0.5 (0, 00032)
F3	0.27 (0, 00018)	0.2 (0, 0004)

### 7.2. Application to the HDSN Coverage Problem 

#### 7.2.1. Experimental Setup

In this section, the performance of the proposed algorithm is analyzed and the parameters of the algorithm are the same as the previous section in performance evaluation in test functions. The following network parameters are set: *m* = *n* = 40, *N* = 300, sensor parameters are presented in Table 4.

**Table 4:** Sensor specifications.

Type	Sensing Radius	Radius Communication	Angle of View	Quantity
1	10	20	π/3	150
2	15	30	π/2	150

**Figure 3:**
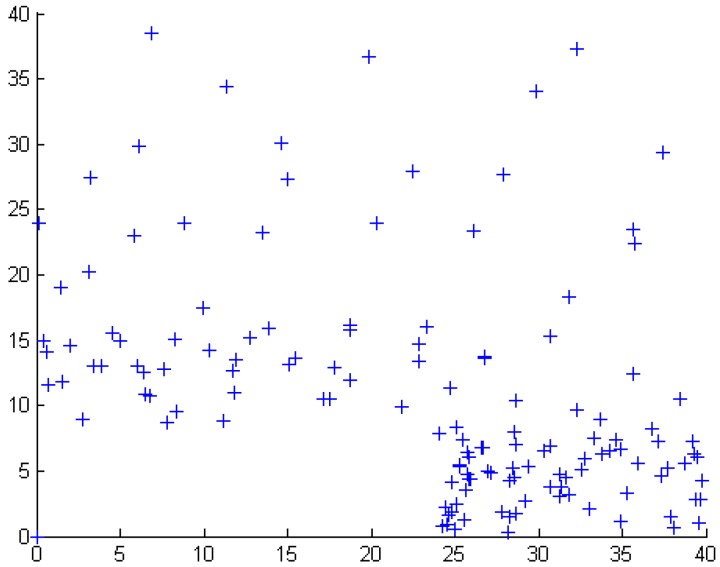
Clustered layout of targets (150 targets in a 40 × 40 area).

In order to model the hotspots, we divide the monitor area into a number of squared subareas. For each subarea, we assign a number of targets to that subarea according to a bounded Pareto distribution. A subarea with a lot of targets can be viewed as a hot spot. Once the number of targets in a specific subarea is determined, they are randomly distributed within that subarea. The cumulative density function of the bounded Pareto distribution is:

$$F(k)=\frac{1-{\left(u/k\right)}^{\alpha }}{1-{\left(u/v\right)}^{\alpha }}$$

where *u* < *k* < *v*, 0 < α < 2 [40]. In this paper, *u* = 3, *v* = 100, and α = 1.1, the number of targets and subareas are 150 and 36, respectively. Figure 3 illustrates the targets distribution of the clustered layout generated by the above-mentioned method. In our experiment, the parameter *K*, that is coverage requirement and connect requirement is in the hot area, which is set 2, otherwise, *K* = 1 in the non- hot area.

#### 7.2.2. Experimental Results and Analysis

**Figure 4:**
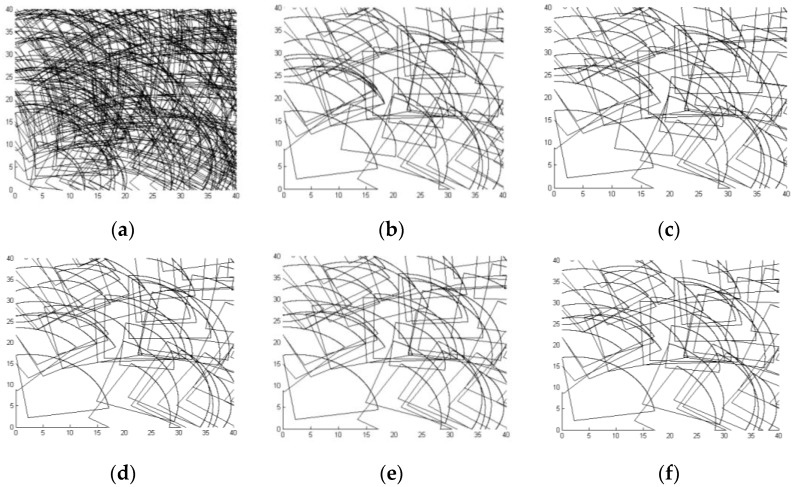
One of the non-dominated solutions. (**a**) 300 nodes, initial distribution; (**b**) the 100th generation, 66 nodes, 89.3% coverage rate, full connect value; (**c**) the 200th generation, 62 nodes, 90.9% coverage rate, full connect value; (**d**) the 400th generation, 58 nodes, 91.3% coverage rate, full connect value; (**e**) the 600th generation, 57 nodes, 92.9% coverage rate, full connect value; (**f**) the 800th generation, 53 nodes, 95.8% coverage rate, full connect value.

The results generated by LACRO with different generations are illustrated in Figure 4. As can be seen from the figure, with the increase in the iteration number, the solutions obtained by LACRO approximate optimal, which verify the effectiveness of the algorithm in solving multi-objective coverage optimization problems in HDSNs. 

Experiment 1: Fitness comparison

**Figure 5:**
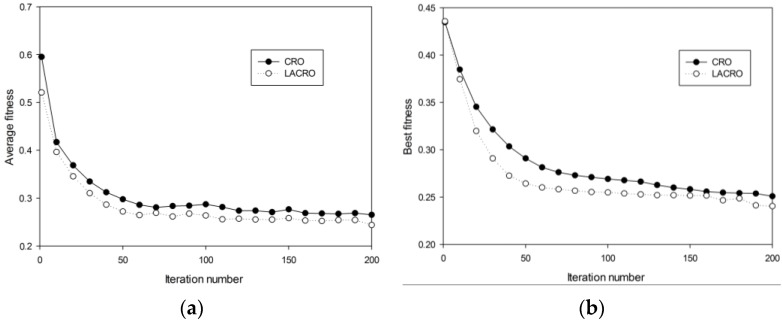
(**a**) Average fitness in various generations; (**b**) Best fitness in various generations.

This experiment studies the exploration/exploitation capabilities of the algorithms. Figure 5. shows the evolution of the average fitness and better fitness with the iteration number. As can be seen in the figure, the two curves are noticeably different and the LACRO curve converges more rapidly and effectively than the CRO curve, which is strong proof that LACRO has better performance than CRO. 

Experiment 2: Coverage control performance comparison

**Figure 6:**
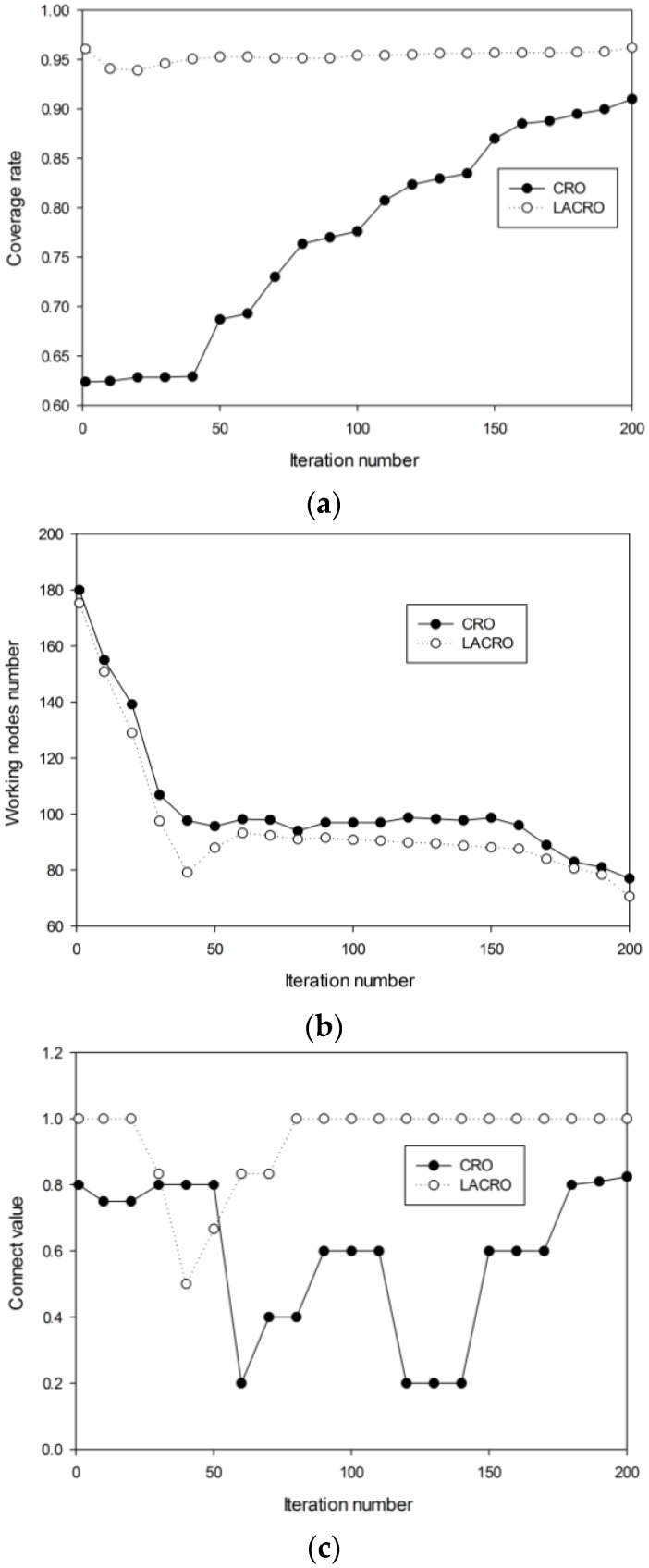
(**a**) Coverage rate *f_1_* in various generations; (**b**) Ratio of working nodes *f_2_* in various generations; (**c**) Connect value *f_3_* in various generations.

In order to compare the performance of the two algorithms in the HDSN multi-objective optimization problem, several experiments are carried out and the results are presented in Figure 6. As can be seen from the figure, LACRO can converge more rapidly and obtains better solutions than the CRO algorithm in coverage rate, working node number and connect value. Due to the random nature of the CRO algorithm, it may “vibrate” in its optimization process as can be seen from Figure 6, especially in Figure 6c. Overall, we can claim that LACRO outperforms CRO in these problems.

Experiment 3: Performance evaluation between LACRO and other algorithms

**Figure 7:**
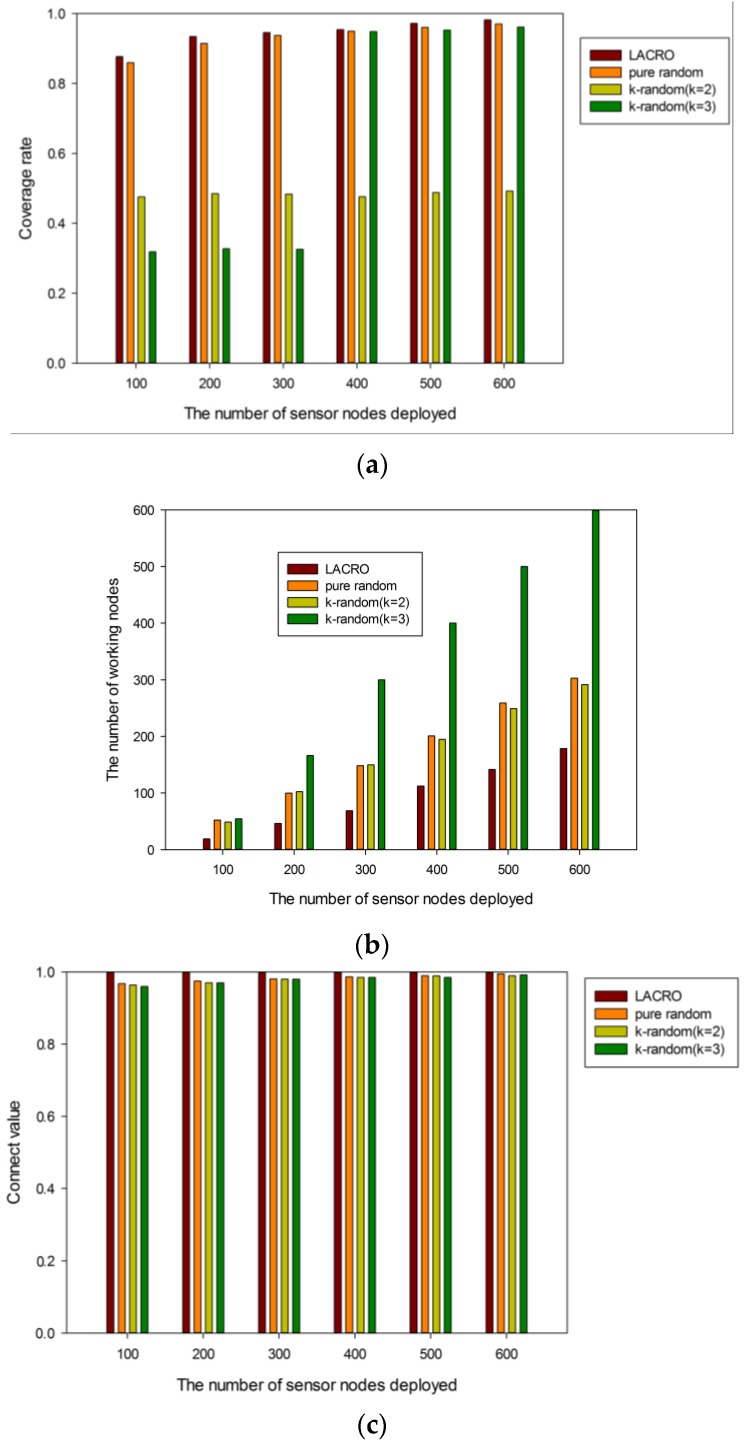
(**a**) Coverage rate *f_1_ vs.* node density; (**b**) Ratio of working nodes *f_2_ vs.* node density; (**c**) Connect value *f_3_ vs.* node density.

In order to compare the performance of different approaches, we run the pure random algorithm and k-random algorithm [41] where *k* is equal to 2 and 3, respectively. The results are illustrated in Figure 7. We can see clearly that LACRO needs the least amount of sensor nodes to ensure full connectivity value and the best coverage rate among the comparison algorithms. Thus it is fair to claim that LACRO has better optimization ability and scalability compared to other coverage control algorithms and it is much more effective for solving multi-objective coverage control problems with large dimension sizes. 

## 8. Conclusions and Future Work

Featured by the geographical irregularity of the sensed events and heterogeneity of the sensor nodes, this paper presents a novel multi-objective optimization problem of HDSN which guarantees the coverage rate of the monitor area, the financial cost of deployment and the directional sensor network communication connectivity. Our proposal is a learning automata-based coral reef optimization algorithm (LACRO) which equips the CRO with the ability to create spur-in-time responses with better exploration/exploitation capabilities as a result. The CRO parameters are selected by the learning automata. The parameters of *F_b_* and *B_d_* are discretized within their permitted ranges, and a learning automaton with a finite action set is used for each parameter. An enhanced decomposition method based on the Tchebycheff Approach is proposed to decompose the multi-objective problem into some single-objective problems. Experimental results indicate that the proposed algorithm and the improved Tchebycheff method are highly competitive with the CRO algorithm in test functions and multi-objective optimization problems in HDSNs. In the future, we will study energy efficient coverage optimization algorithms in mobile HDSN, which can adjust the working direction of the nodes and change their physical positions to improve the network coverage. 
